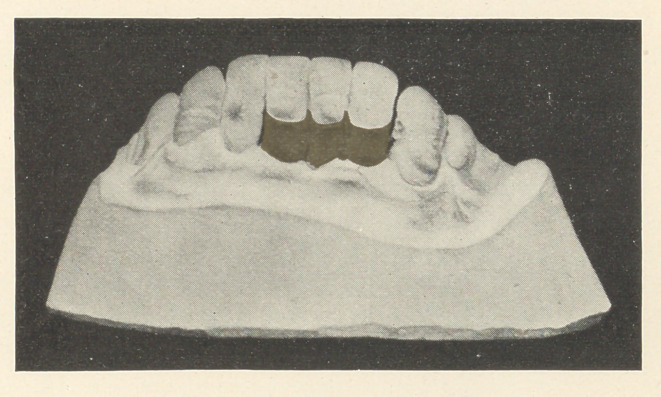# Securing Loose Teeth Affected with Pyorrhœa

**Published:** 1899-07

**Authors:** L. C. Bryan

**Affiliations:** Basel, Switzerland


### SECURING LOOSE TEETH AFFECTED WITH PYORRHŒA.[1]

BY DR. L. C. BRYAN, BASEL, SWITZERLAND.

THERE are often cases of pyorrhœa loosening lower incisor teeth, and various methods have been recommended for securing these in place and for strengthening them. The usual method of procedure is to cut a groove in the cutting edge of the teeth and insert a gold bar, with little pins soldered to the bar and running into the point of each tooth. These are secured with cement or gold filling round the edges. The first difficulty in this method is

[1] Read before The New York Institute of Stomatology, April 4, 1899.

as regards the bite, which is almost always close, and the teeth have to be cut to make room for the bar. The next objection is the serious one of disfigurement of the mouth, with a shining gold bar extending along the cutting edge of the incisors.

Having a case some years ago of this kind of diseased teeth, where the two centrals were loosened by a standing discharge of pus and were about to be extracted, I decided, instead of doing this, to fit a gold band onto a pure gold plate around the two centrals and the two laterals, in shape something like a double figure eight, extending from the gum margin up on the enamel of the tooth, but not so high as to show in the natural movement of the lips. This band of thin gold was fitted around the necks of the teeth, and with physic forceps was drawn in front and back so as to almost touch between the teeth. Pure gold wire was cut and bent into U-shaped staples. One end of the wire was drawn over the gold plate and the other end under it, extending far enough forward towards the lips to bend the two ends together, and have them just meet when cut off.

The accompanying model is an illustration of a case where three teeth were banded together in this way. In this it will be seen that the wire has been pulled as far as possible forward, passing up at the back in the groove in the plate between each pair of teeth, the ends in front being bent up in a groove in the plate in front from above and below, and cut off so as to meet in the centre. It is necessary to use pure gold for this wire, as any alloy would be somewhat springy, and would not remain where placed.

Before the final bending of this wire the band and wire are fitted on accurately, the wires being then removed.

The gold plate is bent out again from the teeth, and everything being kept dry either with the rubber dam or by painting the margin of the gum with iodine, soft cement is forced between the teeth and the whole inside of the plate, so that when the wires are again put in place and drawn tight the superfluous cement is pressed out above and below the band, and perfectly secures it in position. Lined as it is with cement round the necks of the teeth, they are protected as perfectly as with a gold crown filled with cement before setting would be.

The case here illustrated has had the band in use for four years, and the teeth are perfectly in place, and have grown firm, so far, at least, as pyorrhœa teeth can be firm, and the discharge has long ceased. However, if the band were removed the teeth might still be found to be loose, though the gums have grown up to the band and are firm and healthy.

This gold band is worn with the greatest comfort, tartar from the sublingual glands filling in spaces from the back and requiring to be cleaned and polished, as the other lower incisors are, with scalers.

This method had given so much satisfaction that I have used it in several cases where I have removed gold bars and replaced them with this invisible gold band. By reference to the model the *modus operandi* will be clearly seen, and many points not clear in the text will be elucidated. The band I set with Weston's insoluble cement, which I also use for setting crowns instead of Weston's crown cement. There may be other cements equally good for this purpose and for setting crowns, but owing to the fact of it being possible to use this at a creamy consistency and of its setting under water or saliva, and having cases where particles of this cement have remained undissolved about the edges of crowns after years of exposure to the serum and fluids exuded from the gum margin, I have used Weston's cement in preference to any other.

Just here let me say that our profession would make a good deal more progress if dentists were a little less fearful of publishing their experiences and of recommending articles which have proved successful in their hands, and also publicly condemning those which have been found wanting.

## Figures and Tables

**Figure f1:**